# Nerve Diseases

**Published:** 1894-03-10

**Authors:** 


### Medical Progress.

### NERVE DISEASES.

*Puncture, for Diagnostic and Therapeutic Purposes.*—Ziemssen[14] records the experience of himself and others in puncturing the spinal canal, both as a therapeutic and diagnostic measure, originally recommended by Quincke; temporary relief is all that has been obtained hitherto. The amount of albumen would appear to be capable of determining whether the effusion is an inflammatory or simply dropsical exudation.

*Syringomyelia.*—The pathological conditions associated with this disease seem to become more indefinite rather than more precise. Dr. Beevor[15] described at the Clinical Society of London a case in an adult man, who during life had had loss of sensibility to pain, heat, and cold over the whole of the right leg, right half of the trunk up to the fourth rib, while tactile perception was normal. There was also muscular wasting of both upper limbs, wasting of left serratus magnus, and great weakness of the left inferior extremity. The loss of heat sensibility without impairment of tactile sensibility led to the diagnosis of syringomyelia. On post-mortem examination, however, two syphilitic tumours were found—one on either side of the cervical enlargement, causing pressure upon the cord from without. Dr. Eskridge[16] narrates a case occurring in a middle-aged man, in which in the course of four years there had developed complete loss of tactile sense on the left side up to the median line from the crown of the head to the sole of the foot, and a very considerable diminution of tactile sensibility upon the right side; temperature sense and pain sense appear to have been entirely absent, firm pressure upon the testicle, for example, causing no unpleasant sensation; pressure sense, taste, and smell were also almost in abeyance, hearing power diminished, and the fields of vision greatly narrowed. There was muscular weakness and wasting on the left side, the deltoid being specially affected. On these last grounds Eskridge excluded hysteria as a cause, and thinks the probable lesion is a growth of gliomatous structure in the gray matter of the cord and medulla, one of the conditions to which the name of syringomyelia has been given. C. L. Dana[17] brought forward at the New York Neurological Society the case of a man who had a central gliomatous tumour in the lower dorsal region of the cord. Among other symptoms there had been anæsthesia of the right side, extending up to the twelfth dorsal spine, involving sensibility to touch temperature, and pain. In commenting upon the case, Dana said he had come to agree with the psychologists that pain was not a sensation, but a form of feeling; that it was not to be classed with the sensations of touch and temperature; that it did not have peripheral end organs, and that there was no such thing as a pain tract in the cord. To mix up pain-sense, touch-sense, heat-sense, &c., was to show a mental confusion unworthy of advanced neurologists. Pain was much more closely allied to hunger than to touch. In the discussion it was generally considered that clinical evidence strongly supported the view of there being a pain sense; even, if physiologically, it was not certain that it was transmitted up the cord in the antero-lateral columns. Ehlers, of Copenhagen,[18] gives an interesting discussion upon the relationship which Zambaco believes to exist between leprosy, Morvan's disease, and syringomeylia. It is obvious that this last term must now be taken to comprise a variety of spinal disorders, irrespective of the point whether there be an anatomically true syringo-myelosis or not.

*Peripheral Neuritis.*—The relations etiological and pathological of peripheral neuritis become more complicated. Dr. A. W. Campbell[19] records four fatal cases of alcoholic neuritis. Besides the usually recognised local lesions, all presented disease of spinal cord and medulla, viz., a scattered degeneration in all the white columns, the columns of Goll and Burdach and Lissauer's root-zones having suffered in a marked degree. The degeneration did not follow physiological lines, and motor and sensory columns were alike alike affected. The nerve-roots were also degenerated. Dr. J. J. Putnam, of Harvard,[20] also records a fatal acute case of polyneuritis in which poliomyeletis was found also, and still another case in which foci of encephalitis were found along the walls of the third ventricle. Incidentally J. J. Putnam remarks that in children lead-palsy is as likely to affect the legs as the arms. Upon the question of etiology he remarks that occasionally the cause of an attack remains undiscoverable after lead, arsenic, diphtheria, and alcohol have been ruled out. Multiple neuritis occurs in epidemics among the fishermen of the Atlantic seaboard of the States from an unknown cause. Dr. Mott[21] demonstrated at the Clinical Society of London a case of multiple toxæmic neuritis occurring in an adult man of unknown origin. Buzzard[22] records case of multiple neuritis occuring from the administration of Fowler's solution in considerable doses for the treatment of congenital chorea; the symptoms lasted several months. Lamy[23] observes that three forms of neuritis follow childbirth; two are of local origin, the third affects the upper as well as the lower extremities, is infectious, and is the homologue of the neuritis following small-pox and other such diseases. Clinically there is a great resemblance to acute central myelitis. J.W. Putnam, of Buffalo,[24] calls attention to need of rest and sedatives in earliest stage of neuritis, afterwards to gentle stimulation by mild galvanic current and manipulation of joints, which often get stiff if not kept moved. Bollenstern[25] records a very severe case of alcoholic neuritis cured in four weeks by hypodermic injection of 1 per cent. solution of strychnine in increasing doses from one minim up to ten, with intermissions of a few days from time to time.

*Sciatica.*—Weir Mitchell,[26] in obstinate chronic cases of sciatica, envelopes with a flannel bandage the whole of the affected limb, and a splint, in such a fashion as to keep the hip and knee immovable. The bandage of pure flannel is applied twice a day, the leg being kept slightly bent at the knee and extended at the thigh, and in this position it is secured to a splint which passes from the axilla to the ankle. Three weeks of uninterrupted treatment are necessary, then the bandage and splint are gradually left off. Dr. J. E. Atkinson,[27] desiring to avoid the hypodermic use of morphia for relieving the pain of sciatica, recommends injecting fifteen minims of sulphuric ether every other day along the course of the nerve. Sloughing has been known to occur after this mode of treatment.

*Vertigo.*—C. L. Dana[28] divides vertigo into these varieties: (1) The "vascular" form, dependent upon hæmic and cardiac causes; may be simply anæmic or toxæmic; (2) "auditory," due to disturbance of the space-sense branch of eighth nerve; the severest and most typical form, known as Ménière's vertigo, is due to organic disease of the middle ear, but there are many milder forms of functional origin; (3) "ocular," arising from disturbance either in refraction or in the muscles of the eye, such as astigmatism or muscular asthenopia, but these cases are rare; (4) due to reflex irritation from abdominal and pelvic viscera, the most common form; (5) neurotic, *e.g.*, the epileptic and neurasthenic forms.

*Cerebellar Ataxy.*—Dr. Ashby,[29] in the course of an excellent article on "Diagnosis of Cerebellar Tumours," states that he doubts the existence of any special cerebellar ataxy, but the gait, like that of a child learning to walk, is characterised by clumsiness and readiness to fall, this, however, being due to weakness of the limbs and not to ataxy. The *Lancet*,[30] commenting upon this idea of Dr. Ashby's, believes him to be mistaken, and that, whether spasticity is present or absent in these cases, there may be a reeling gait independent of the presence or absence of spasticity and also of weakness.

*Cerebral Peduncle, Tumour of, Accompanied by Tremor.*—Blocq and Marinesco[31] reported at the Biological Society of Paris a case in which during life there had been upon the left side tremor indistinguishable from that of paralysis agitans; at the autopsy a tumour the size of a gooseberry was found in the substance of the right cerebral peduncle involving the greater part of the locus niger, but the root of the cerebral peduncle, the superior peduncle of the cerebellum, and the second nerve were not affected. Charcot, Mendel, and Benedikt have observed similar cases, but the pathogeny of the tremor is obscure.

*Hypnotics and Analgesics.*—Dr. Gordon Sharp[32] states that he considers hyoscine hydrobromate in its clinical effects to resemble in every way those of atropine, and not to be a motor calmative and hypnotic in delirium. It cannot, in his opinion, be recommended as a safe hypnotic. Dr. W. P. Spratling[33] records the effects of hyoscyamin upon himself; there was no tendency to sleep, motor paralysis supervened, beginning with the lower extremities and passing upward; grosser movements suffered first, motor and sensory paralysis lasted about four hours, but at no time was there either sleep or unconsciousness, only delirium and delusions. Dr. Spratling believes that the hypnotic influence of hyoscyamin and hyoscine is only apparent, being solely due to the motor paralysis they cause. Duboisin administered hypodermically where morphia had failed in the treatment of hystero-epilepsy, is praised by Albertoni,[34] also independently by both Belmondo and Samuely[35] in doses of from one half to two milligrammes. Phosphate of soda administered hypodermically in locomotor ataxy, neurasthenia, and hysteria is believed by Tonolli[36] to be of utility. It has been employed also in Belgium and in this country by

L. F. Winslow, but has not obtained confidence generally. Coombemale[37] gives results of a series of experiments to ascertain whether exalgine possessed any local anæsthetic power. He concludes that it has a definite anæsthetic action, small in degree and transitory, but possibly sufficient for momentary operations and explorations. Edward Younger,[38] in opposition to views of Montyel, has found exalgin useful in cases of mental disease associated with subjective symptoms similar to those of neuralgic disorders. Collatz[39] contributes statistics to show value of trional as a remedy for sleeplessness in mental disease. Thirty grains is the ordinary dose, but two drachms taken with suicidal intent were not finally injurious. Without being absolutely certain, trional is free from injurious effects, and is suitable for prolonged administration. F. G. Morrill[40] states he has given chloralose with good results in almost every instance. A dose of three grains, repeated if necessary in three hours, will secure five to nine hours of refreshing sleep. In locomotor ataxy, somnambulism, and pulmonary phthisis the conditions are considered to be unfavourable to its action.

*Organic Extracts* are still being employed as nervine tonics. Julius Althaus[41] reports upon the subcutaneous use of a glycerine preparation of the brain and of the spinal cord, under the names of alpha-cerebrine and alpha-myeline respectively, as a remedy in nerve disease. The general result at which he arrived is that these substances are no specific remedies for disease of the nervous system. They are useless in epilepsy, paralysis agitans, and tremor, as well as in the usual forms of hemiplegia. On the other hand, he is convinced that they constitute a nervine tonic of considerable efficacy in certain maladies and conditions mainly characterised by loss of nerve-power. He records several cases of locomotor ataxy, spastic spinal paralysis, senile debility, and other conditions which improved under the mode of treatment. Prof. Alex. Robertson[42] believes that he has seen distinct results resulting from the administration *per os* of a glycerine extract of sheep's brain in some cases of melancholia, and in one case of chronic myelitis. He thinks that there is a constituent in the brain of the sheep which acts as a stimulus to nerve tissue, cell and fibre, in the human subject, but that probably more decided beneficial effects would follow subcutaneous injection of the extract. Dr. R. Evans,[43] of Selkirk, reports a case of neurasthenia benefited by hypodermic injection of Constantin Paul's nerve extract. Alombert-Goget[44] says that Brown-Sequard's fluid is of some service in ataxy and delirious epilepsy, causing an increase of vitality in every respect; and Cullère[45] states that it has an almost immediate stimulating function on deranged persons, as well as upon tuberculous subjects; the psychopathic state is often ameliorated, but a permanent improvement has not been obtained.

[The orchitic and brain fluids, as obtained in this country from Paris, have yielded no appreciable result in our hands in cases of various decriptions.—ED.]

[14] Wien. Med. Presse, quoted in Annals of Surgery, Sept., 1893. [15] Med. Week, Nov. 17, 1893. [16] Internat. Clin., Vol. III., 1893. [17] N. Y. Med. Journ., Dec. 23, 1893. [18] Med. Week, Sep. 29, 1893. [19] Liverpool Medico-chirurg. Journ., quoted in Lancet, Dec. 16, 1893. [20] International Clinics, Vol. IV. [21] Lancet, Dec. 2, 1893. [22] Lancet, Nov. 18, 1893. [23] Arch. de Tocol. et de Gynec., quoted Ep. B. M. J., Jan. 20, 1894. [24] International Clinics, Vol. IV. [25] Therap. Monats, No. 8, 1893, quoted in Clin. Journ., Jan. 10, 1894. [26] Philadelph. Med. News, quoted in Lancet, July 29, 1893. [27] Internat. Clinics, Vol. IV., 1893. [28] Internat. Clinics, Vol. IV. [29] Practitioner, Dec., 1893. [30] Dec. 23, 1893. [31] Med. Week, June 2, 1893. [32] Practitioner, Jan., 1894. [33] N. York Med. Rec., Dec. 16, 1893. [34] Ep. Brit. Med. Journal, March 25, 1893. [35] Ibid. [36] Ep. Brit. Med. Journ., Dec. 30, 1893. [37] Bulletin Médical du Nord, quoted in N. Y. Med. Rec., Dec. 23, 1893. [38] Bullet. Gén. de Thér., quoted in Therap. Gaz., p. 841, 1893. [39] Berl. Klin. Woch., quoted in Practitioner, Dec., 1893. [40] Boston Med. and Surg. Journ., quoted in Ep. B. M. J., Dec. 23, 1893. [41] Lancet, Dec. 2, 1893. [42] Brit. Med. Journ., Dec. 16, 1893. [43] Ibid. [44] Lyon Méd., Nov. 12, 1893. [45] Gaz. Méd. de Paris, quoted in Therap. Gaz., April, 1893.

